# Mechanical Regulation of Redox Balance via the Induction of the PIN1/NRF2/ARE Axis in Pancreatic Cancer

**DOI:** 10.3390/ijms24043476

**Published:** 2023-02-09

**Authors:** Chen Liang, Zeyin Rong, Abudureyimu Tuerhong, Qingcai Meng, Jie Hua, Jiang Liu, Bo Zhang, Wei Wang, Xianjun Yu, Si Shi, Jin Xu

**Affiliations:** 1 Department of Pancreatic Surgery, Fudan University Shanghai Cancer Center, Shanghai 200032, China; 2 Department of Oncology, Shanghai Medical College, Fudan University, Shanghai 200032, China; 3 Shanghai Pancreatic Cancer Institute, Shanghai 200032, China; 4 Pancreatic Cancer Institute, Fudan University, Shanghai 200032, China

**Keywords:** pancreatic cancer, mechanical force, redox balance, PIN1, NRF2

## Abstract

Pancreatic cancer is one of the most lethal malignancies. Desmoplastic stroma and metabolic reprogramming are two hallmarks of pancreatic cancer that support its malignant biological behaviors. However, the underlying mechanism by which the stroma maintain the redox balance remains unclear in pancreatic ductal adenocarcinoma (PDAC). Here, we demonstrated that the physical properties of the stroma could regulate the expression of PIN1 in pancreatic cancer cells. Moreover, we found that hard matrix-cultured pancreatic cancer cells induced the upregulation of PIN1 expression. Since PIN1 maintained redox balance via synergistic activation of NRF2 transcription, PIN1 promoted the expression of NRF2 to induce the expression of intracellular antioxidant response element (ARE)-driven genes. Consequently, the antioxidant stress ability of PDAC was increased, and the intracellular level of reactive oxygen species (ROS) was decreased. Thus, PIN1 is expected to be an important target for the treatment of PDAC, especially PDAC with an exuberant desmoplastic stroma.

## 1. Introduction

Pancreatic ductal adenocarcinoma (PDAC) represents the most common form of pancreatic cancer, and is the third leading cause of death in men and women with an overall 5-year survival rate of less than 9% [1,2]. This dismal prognosis is primarily attributed to the observation that this cancer has usually developed into locally advanced or metastatic disease at the time of diagnosis, and thus, fewer than 20% of patients have localized potentially curable tumors [1,3]. Even after curative surgery at early stages, local and metastatic recurrences occur, making PDAC highly resistant to any therapeutic regimen [4,5]. Therefore, it is of utmost importance to identify the mechanisms underlying PDAC progression and to develop novel treatments that can improve the prognosis of this disease.

In PDAC, interactions between neoplastic and nonneoplastic cells within the extracellular matrix (ECM) have been proposed to stimulate the extensive desmoplastic reaction that is responsible for the main tumor bulk and accounts for up to 90% of the tumor volume. Thus, compared with other malignancies, a cardinal histopathological feature of PDAC is the occurrence of prominent hyperplasia of the stroma surrounding the local infiltrated tumor tissues that distorts the normal architecture of the pancreatic tissue [6,7]. The desmoplastic stroma makes the tumor hard and provides mechanical cues to the cells that trigger biochemical signals through mechanotransduction. One such cue is the stiffness of the material. Cells sense this stiffness primarily through integrin-mediated and cadherin-mediated adhesions that couple the extracellular matrix and the environment of the interacting cells to the actin cytoskeleton. PDAC are often stiffer than normal tissues and show abnormally fast metabolism of glucose [7,8,9]. The link between these two traits involves tension in a network of protein filaments within cells. In turn, the tumor microenvironment- and genotype-specific metabolic adaptations reprogram cancer cells and the microenvironment to promote the tumorigenesis. In addition to providing energy and building blocks, the main consequence of metabolic reprogramming is to defend against oxidative stress to maintain the redox balance [7].

Nuclear factor E2-related factor 2 (NRF2) is a leucine zipper transcription factor to maintain the cellular redox homeostasis [10]. NRF2 binds to a specific DNA sequence, referred to as the antioxidant response element (ARE), and it induces the expression of a cohort of antioxidant-related genes. NRF2 and the ARE-driven genes are frequently upregulated in PDAC and are correlated with poor survival [11,12]. Previous studies have shown that protein interacting with never in mitosis A1 (PIN1), a member of the parvulin subfamily of peptidyl prolyl cis/trans isomerases (PPIases) catalyzes the isomerization of ERK-phosphorylating NRF2 to activate it, synergistically maintaining the redox balance in PDAC by upregulating the expression of ARE-driven genes [13,14].

Here, we show that PIN1 expression is positively correlated with tumor stiffness and the stromal proportion in PDAC. Given the oncogenic effect of PIN1 on pancreatic tumorigenesis, we demonstrate that mechanical stress can upregulate PIN1 expression to decrease the reactive oxygen species (ROS) level and defend against oxidative stress. Our study identified mechanical stress as an extracellular factor that maintains redox homeostasis by regulating the PIN1/NRF2/ARE axis, thereby highlighting PIN1 as an important target for the treatment of PDAC, especially PDAC with an exuberant desmoplastic stroma.

## 2. Results

### 2.1. PIN1 Expression Is Positively Correlated with the Stromal Content of the PDAC

**Figure 1:**
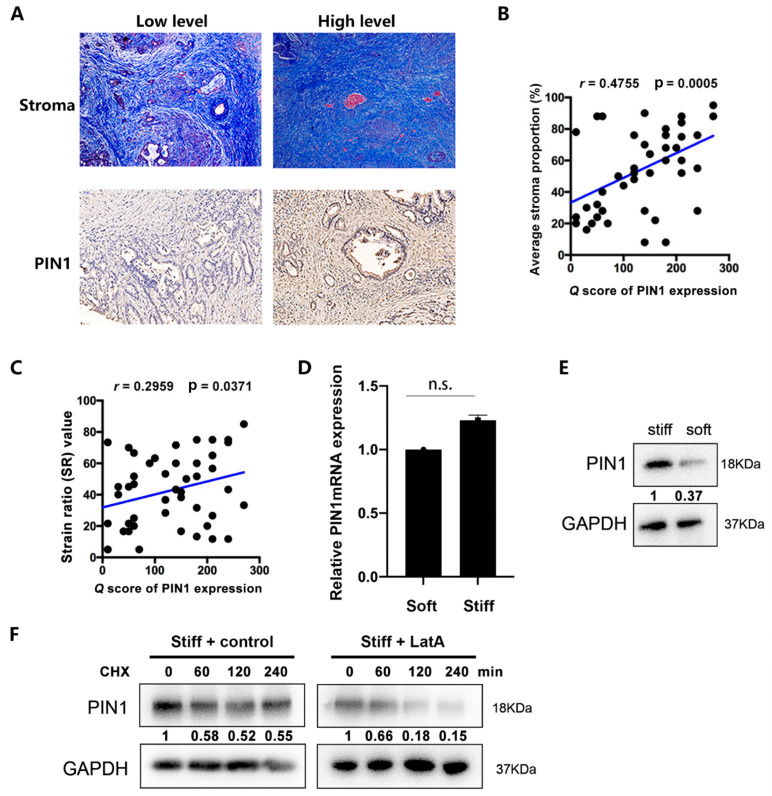
PIN1 expression is positively correlated with the stromal content in PDAC. (**A**) Representative image (magnification, ×100) of PIN1 expression and stroma proportion in serial sections of human pancreatic cancer tissues from the FUSCC cohort, as analyzed by immunohistochemical staining and Masson’s trichrome staining, respectively. (**B**) Positive correlation between PIN1 expression and stroma proportion in PDAC samples (Spearman r = 0.4755, *p* = 0.0005). (**C**) Positive correlation between PIN1 expression and SR value correlation of the stromal area with PIN1 ex-pression in PDAC samples (Spearman r = 0.2959, *p* = 0.0371). (**D**) The mRNA expression levels of PIN1 in the PANC-1 cells cultured with matrices with different stiffnesses, as determined by qRT-PCR. Data are expressed as the mean ± SD of three independent experiments, n.s. = not statistically significant. (**E**) Western blotting of lysates from PANC-1 cells cultured with matrices with different stiffnesses by the antibody against PIN1. (**F**) PANC-1 cells cultured with stiff substrate were treated with LatA (200 nM) or vehicle and then were treated with cycloheximide (CHX, 30 mg/mL) for various time intervals. Cell lysates were then analyzed by immunoblotting with indicated antibodies.

PDAC is a malignant tumor rich in stroma. To identify the effect of the stroma on PIN1 expression, we analyzed the stromal content by Masson staining and the expression level of PIN1 by immunohistochemical staining of samples from 50 patients with PDAC in the FUSCC cohort (Figure 1A). The results indicated that upregulation of PIN1 expression was correlated with a dense stroma (Figure 1B). Our previous study has shown that the dense stroma always displayed the high stiffness. Thus, we detected the strain ratio (SR) values, which provide a quantitative measure of tumor stiffness in pancreatic cancer, by endoscopic ultrasonography. As shown in Figure 1C, PIN1 expression was positively correlated with the SR values, suggesting that stiffer PDAC has a higher level of PIN1 expression.

To simulate the soft and stiff mesenchymal environment in vivo, we constructed a soft and stiff cell culture microenvironment through applications of soft and stiff substrates. It was found that a stiff matrix culture environment could induce the upregulation of PIN1 expression at the protein level, but there was no significant change in the PIN1 mRNA level (Figure 1D,E). This finding suggests that the microenvironment has no effect on the transcription of PIN1 but might regulate the protein stability of PIN1. Thus, we used cycloheximide (CHX) to examine the half-life of the PIN1 protein. The results indicated that a stiff substrate could affect the cytoskeleton to increase the protein stability of PIN1 by treatment with latrunculin A (LatA), one actin-monomer-sequestering compound (Figure 1F), suggesting that cytoskeleton system is likely involved in the upregulation of PIN1 by stiff microenvironment.

### 2.2. Stiff ECM Improves Oxidative Stress in Pancreatic Cancer Cells

**Figure 2:**
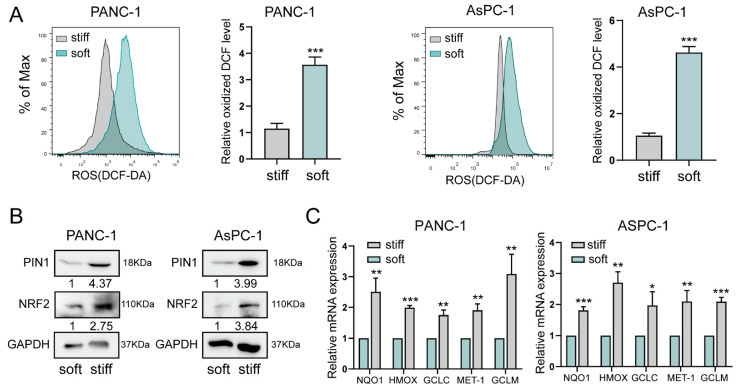
Stiff ECM improves oxidative stress in pancreatic cancer cells. (**A**) Analysis of ROS production by flow cytometry in PANC-1 and AsPC-1 cells grown on the substrates with different stiffness (left panel). Quantification of ROS production is shown as the mean ± SD of three independent experiments; ***, *p* < 0.001 (right panel). (**B**) Western blotting of lysates from PANC-1 and AsPC-1 cells cultured with matrices with different stiffnesses by the indicated antibody. (**C**) The mRNA expression levels of ARE-driven genes in the PANC-1 and AsPC-1 cells cultured with matrices with different stiffnesses, as determined by qRT-PCR. Data are expressed as the mean ± SD of three independent experiments; * *p* < 0.05; ** *p* < 0.01; and *** *p* < 0.001.

Because our previous study found that PIN1 was closely related to the maintenance of redox balance in pancreatic cancer, we detected the level of reactive oxygen species (ROSs) in pancreatic cancer cells [13]. It was found that the level of ROSs in pancreatic cancer cells PANC-1 and AsPC-1, which were grown on a stiff substrate, were significantly downregulated (Figure 2A). NRF2 is an important transcription factor to induce the expression of a cohort of antioxidant-related genes, and thereby it plays a key role in maintaining the cellular redox homeostasis. Previous study has shown that there was a positive correlation between PIN1 and NRF2 in patient samples by IHC staining. Further studies showed that this phenomenon might be related to the upregulation of NRF2 expression induced by PIN1 in hard matrix-cultured pancreatic cancer cells (Figure 2B). We also examined the expression of ARE-driven genes (GCLC, GCLM, ME-1, HMOX, and NQO1) and shown that stiff microenvironment also induced the expression of a series of antioxidant molecules downstream of NRF2 (Figure 2C).

### 2.3. Stiff ECM Induced PIN1 Expression to Play Antioxidant Roles

**Figure 3:**
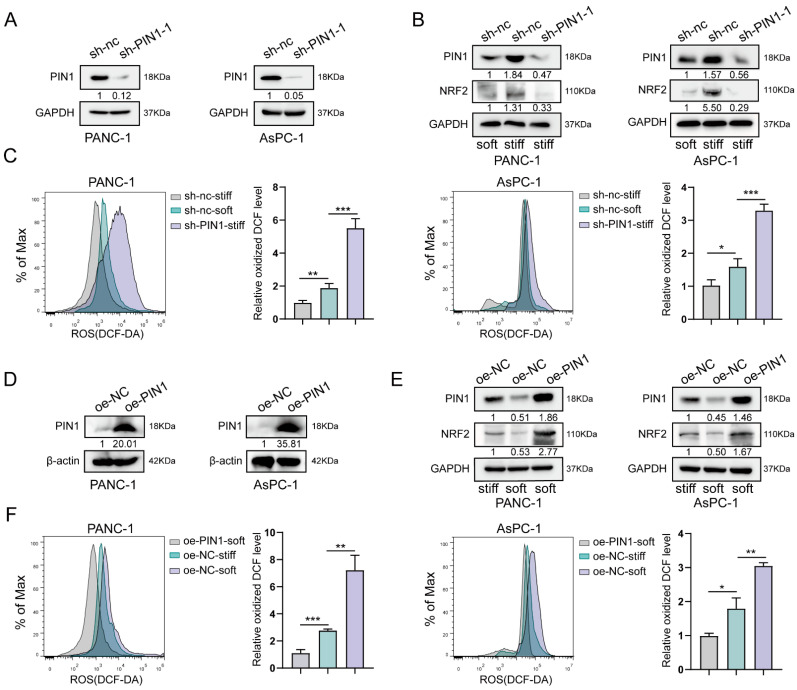
Stiff ECM induced PIN1 expression to play antioxidant roles. (**A**) PIN1 expression was silenced in AsPC-1 and PANC-1 cells by transfection with shRNA against PIN1, as determined by Western blot. (**B**) NRF2 and PIN1 expression in PANC-1 and AsPC-1 cells transfected with PIN1-shRNA and negative control-shRNA, which were cultured on a stiff substrate, as determined by western blot. (**C**) ROS production in cells transfected with PIN1-shRNA and negative control-shRNA, which were cultured on a stiff substrate. Analysis of ROS production by flow cytometry (left panel). Quantification of ROS production is shown as the mean ± SD of three independent experiments; * *p* < 0.05; ** *p* < 0.01; and *** *p* < 0.001 (right panel). (**D**) Western blotting of lysates from PANC-1 and AsPC-1 cells constructed to overexpress PIN1. (**E**) Western blotting of lysates from PIN1-expressing cells cultured on a soft substrate by the antibodies against PIN1 and NRF2. (**F**) ROS production in PIN1-expressing cells cultured on a stiff substrate. Analysis of ROS production by flow cytometry (left panel). Quantification of ROS production is shown as the mean ± SD of three independent experiments; * *p* < 0.05; ** *p* < 0.01; and *** *p* < 0.001 (right panel).

To identify the effects of PIN1 on stiff ECM-induced antioxidation in PDAC, we first silenced PIN1 expression in AsPC-1 and PANC-1 cells by transfecting shRNA against PIN1 (Figure 3A). Furthermore, compared with cells cultured on a soft substrate, we examined the NRF2 expression in cells transfected with PIN1-shRNA and negative control-shRNA, which were cultured on a stiff substrate. The results indicated that silencing PIN1 could inhibit the stiff ECM-induced upregulation of NRF2. The level of NRF2 protein in cells with silencing PIN1 was similar to that in PANC-1 and AsPC-1 cells cultured on a soft substrate (Figure 3B). Moreover, we also found that silencing PIN1 significantly upregulated ROS production to counteract the antioxidative effects of the stiff substrate (Figure 3C). Meanwhile, we constructed pancreatic cancer cells with PIN1 overexpression (Figure 3D), and the results demonstrated that overexpression of PIN1 in cells cultured on a soft substrate could re-activate the upregulation of NRF2 and inhibit the ROS production. These indicated that a stiff substrate could induce PIN1 upregulation to activate the antioxidation of NRF2 to repress ROS production (Figure 3E,F).

### 2.4. The Cytoskeleton Is Involved in Stiff ECM-Induced Antioxidation in Pancreatic Cancer

**Figure 4:**
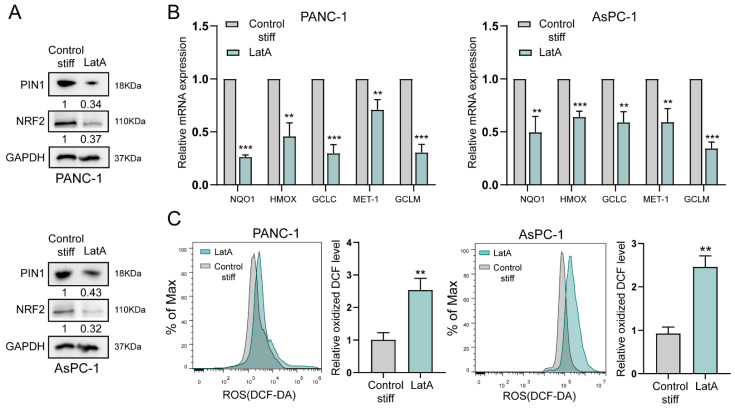
The cytoskeleton is involved in stiff ECM-induced antioxidation in pancreatic cancer. (**A**) Western blotting of lysates from PANC-1 and AsPC-1 cells cultured with stiff substrate and treated with LatA (200 nM) or vehicle by the indicated antibody. (**B**) qRT-PCR analysis of the mRNA expression of ARE-driven genes in PANC-1 and AsPC-1 cells cultured with stiff substrate and treated with LatA (200 nM) or vehicle. Data are expressed as the mean ± SD of three independent experiments; ** *p* < 0.01; and *** *p* < 0.001. (**C**) ROS production in PANC-1 and AsPC-1 cells cultured with stiff substrate and treated with LatA (200 nM) or vehicle. Analysis of ROS production by flow cytometry (left panel). Quantification of ROS production is shown as the mean ± SD of three independent experiments; ** *p* < 0.01 (right panel).

The mechanosensing of tumor cells affects their biological behavior, mainly by the cytoskeleton system. Thus, cytoskeletal proteins are the subcellular basis and they respond to mechanical forces from the ECM. To test this further, we targeted the F-actin structure directly using LatA. We found that LatA treatment effectively repressed the upregulation of PIN1 expression induced by the stiff substrate in PANC-1 and AsPC-1 cells (Figure 4A). Moreover, the expression of NRF2 and its antioxidative downstream genes was significantly downregulated in PDAC cells treated with LatA (Figure 4B). Meanwhile, accompanying the suppression of antioxidants, LatA also counteracted the ROS level maintained by the stiff substrate to upregulate ROS (Figure 4C). Thus, these results suggested that the stiff substrate induced the upregulation of PIN1 expression, likely by the cytoskeleton, to play an antioxidant role.

## 3. Discussion

Pancreatic cancer is a highly malignant tumor of the digestive system. Compared with other malignancies, there is a lack of effective treatment for pancreatic cancer. This requires us to have a deep understanding of the biological characteristics of pancreatic cancer, identify the key pathways affecting its biological activities, and provide effective interventions, which may help to develop new therapeutic strategies for pancreatic cancer.

In addition to energy, the requirement of antioxidants for the survival of cancer cells makes it possible to develop strategies for targeting cancer that involves disrupting the redox balance [15]. The upregulation of ROS level in cancer cells induces the genetic mutation and is involved in the malignant biological behaviors. Accordingly, cancer cells reprogram the metabolism or increase the activity of antioxidant synthesis pathways to counteract the hyperactive ROS machinery, which enables them to survive in an unfriendly microenvironment [7,16,17]. It is well-known that NRF2 predominantly activates the antioxidant mechanism that tightly controls the ROS levels under conditions of stress stimulation [18,19]. Our previous study also showed that PIN1 protected mitochondrial function partially by affecting NRF2 transcription to maintain cellular redox homeostasis. PIN1 maintained a low level of ROS by the ERK/c-Myc/NRF2 axis to avoid apoptosis and support basal mitochondrial respiration [13].

PDAC is characterized by an exuberant desmoplastic stroma (desmoplasia), with stromal components outnumbering pancreatic cancer cells. An important consequence of the abundant fibrotic stroma is the limitation of proper neovascularization of the tumor (hypovascularization) [20]. This dense fibrotic tissue, together with poor vascularization, limits circulatory access, which creates a severe hypoxic, nutrient-poor, and acidic microenvironment, thereby impairing drug delivery, which induces resistance to chemotherapy. The heterogeneous stroma coevolves with tumor cells and influences PDAC initiation, progression, metastasis, and immune surveillance [7,21]. A recent study pointed out that a PIN1 inhibitor could downregulate the proliferation of pancreatic cancer cells, reduce collagen deposition, and inhibit the activation and proliferation of tumor-associated fibroblasts (CAFs) in the tumor stromal microenvironment [22]. Meanwhile, some studies have shown that during the pathogenesis of a variety of inflammatory diseases, a high level of fibrosis was accompanied by the upregulation of PIN1 expression [23,24]. Therefore, we hypothesized that the highly fibrotic stroma in the tumor microenvironment might be an important factor altering the effect of PIN1 in pancreatic cancer cells. In this study, we detected the abundance of stroma in human pancreatic cancer tissues by Masson’s trichrome staining and detected the protein expression of PIN1 in tissues by immunohistochemical staining. The results showed that PIN1 protein expression was high in pancreatic cancer tissues with rich stroma, suggesting that deregulation of PIN1 in PDAC was closely related with tumor microenvironment.

The effects of the microenvironment on tumor cells are biophysical as well as biochemical [9,25]. Previously, we applied endoscopic ultrasound to detect the strain ratio (SR) of pancreatic cancer in the early stage, which can indirectly reflect the physical attributes of the softness and hardness of PDAC and help us to explore the biophysical effect of stoma on tumor cells [26,27,28]. We demonstrated that the content of stroma in PDAC was directly proportional to the SR value. The more abundant stromal content of PDAC always possesses a higher SR value and presents a harder tumor, accompanied by a poor prognosis [28]. In this study, SR values were positively correlated with the abundance of PIN1 in pancreatic cancer tissues, suggesting that hard pancreatic cancer may play a biophysical role in the regulation of intracellular PIN1 expression. Indeed, we found that PIN1 and NRF2 expression was upregulated in PDAC cells cultured with stiff substrate, suggesting the physical effects of ECM on cancer cells are involved in activating the PIN1/NRF2 axis, which represses the production of ROS and maintains the redox balance. Determining the specific target of this process and achieving an effective intervention by remodeling stromal therapy may help decrease the malignant potential of pancreatic cancer. Therefore, it is particularly important to explore the internal mechanism of the physical effect of stroma on pancreatic cancer cells.

Due to its special biological role, tissue mechanics has attracted increasing attention from cancer researchers in recent years. The mechanosensing of tumor cells can support their metabolic plasticity and energy production, thus influencing their biological behavior. Mechanical forces from the ECM affect the cytoskeleton system through the integrin-mediated signaling pathway and then change the morphology, structure, and biological behavior of the cells. The cytoskeleton is a network of protein fibers in cells and is mainly composed of microtubules, microfilaments, and intermediate fibers. Among them, the microfilaments formed by the aggregation of filamentous actin (F-actin) and myosin use chemical energy to generate mechanical movement and then they aggregate into bundles to form stress fibers in response to the stimulation from the extracellular mechanical force, which is a physical signal [29,30,31]. A recent study caught our attention: in normal human epithelial cells, the mechanical forces generated by the stiff extracellular matrix act on the cytoskeleton to promote F-actin to gather into thick bundles. The TRIM21 protein, an E3 ubiquitin ligase, is attached to it and trapped, reducing the free TRIM21 protein in the cytoplasm, thus limiting its ubiquitin ligase activity [32]. Based on this, we speculate that the regulatory mechanism of PIN1 mediated by stromal mechanical forces in pancreatic cancer might involve remodeling of the cytoskeletal system.

Taken together, our findings elucidated the extracellular factors underlying PIN1 dysregulation and emphasized the biophysical roles of the stroma in redox balance and metabolic reprogramming. Thus, a PIN1-induced antioxidant and cellular detoxification program is expected to provide a therapeutic benefit for pancreatic cancer.

## 4. Materials and Methods

### 4.1. Human Tissues and Cell Culture

A total of 50 human primary pancreatic adenocarcinoma tissues were collected from Fudan University Shanghai Cancer Center (FUSCC). The samples were obtained at the time of surgical treatment and immediately snap-frozen in liquid nitrogen and histologically examined in a timely manner. All samples were obtained with informed consent, and the project was approved by the Clinical Research Ethics Committee of Fudan University Shanghai Cancer Center.

The human pancreatic cancer cell lines PANC-1 and AsPC-1 were obtained from American Type Culture Collection (ATCC). The two cell lines were cultured in DMEM at 37 °C and 5% CO_2_. The cell culture media were supplemented 10% heat-inactivated fetal bovine serum. Cell lines were authenticated by DNA fingerprinting and passaged in our laboratory for fewer than 6 months after their receipt.

### 4.2. Tumor Stroma Measurement

Tumor slices were stained using a Masson Trichrome Staining Kit (Sigma, St. Louis, MO, USA), a three-color histological staining kit in which fibrous tissue is stained blue, nuclei are stained purple black, and cytoplasm, muscle tissue, and erythrocytes are stained red. Digital microphotographs (×100) were taken using a BX43/DP73 microscope (Olympus, Tokyo, Japan), and three microscopic fields were randomly selected. The images were analyzed using ImageJ software (version 1.6; National Institutes of Health, Bethesda, MD, USA), and the average stromal proportions were calculated. The detailed operating protocol is also described in our previous studies [28].

### 4.3. Immunohistochemical Staining

IHC staining of paraffin-embedded tissues with antibodies against PIN1 (Diluted 1:200; Proteintech, 10495-1-AP, Tokyo, Japan) was performed and scored to determine the protein expression profiles according to previously described standard procedures [33]. The following expression levels were based on the score obtained by the intensity and percentage of the IHC staining. The intensity was recorded as 0, 1, 2, and 3, referring to negative, weak, moderate, and strong staining, respectively. The percentage of positive cells was recorded from 0 to 100%. The results of staining were scored using the quick (Q) score, which was obtained by multiplying the percentage of positive cells by the intensity. The median values of the Q scores (Q = 150) were used as cutoff points to classify PDAC as “low expression” or “high expression”.

### 4.4. Reactive Oxygen Species Evaluation

One hour before the end of the experimental time, 1 × 10^6^ indicated cells were incubated with 50 mmol/L 2′,7′-dichlorodihydrofluorescein diacetate (H2DCF-DA) from a Reactive Oxygen Species Assay Kit (Beyotime, Shanghai, China) at 37 °C. The cells were then washed, resuspended in ice-cold phosphate buffered saline (PBS), and collected. The fluorescence intensity of DCF, formed by the reaction of DCF with ROS, was monitored with an excitation wavelength at 488 nm and an emission wavelength at 530 nm by flow cytometry (Beckman, Brea, CA, USA).

#### Soft and Stiff Substrates 3D Cell Culture

Briefly, eight parts of chilled bovine collagen type I gel (PureCol, 2.5 mg/mL; Advanced BioMatrix, Carlsbad, CA, USA) was mixed with one part of 10 × PBS and 1 part of NaOH (0.1 N). The appropriate volume of the mixture was spread over the bottom of a 60 mm glass-bottomed dish (Normax) and incubated for 1–2 h at 37 °C to generate stiff substrates. Bovine collagen type I (PureCol, Advanced BioMatrix, Carlsbad, CA, USA) was diluted in PBS to the appropriate concentration (50 μg/mL), and then tiled to the bottom of the tissue culture dishes to produce soft substrates. Before the formal experiment, 5 × 10^5^ PDAC cells were seeded on top of the gels. Once the cells were attached to the gels, appropriate amount of medium was added and cultured for 24 h. The cells were digested using preheated collagenase type I (Gibco, Carlsbad, CA, USA) at 37 °C for 20 min to collect cells from soft substrates.

### 4.5. Generation of Expression Vectors

To stably knockdown PIN1 in pancreatic cancer cells, shRNA sequence targeting PIN1 (5′-GCCATTTGAAGACGCCTCGTT-3′) was cloned into the lentivector vector GV248, which were synthesized by and purchased from Genechem (Shanghai, China). GV248-sh-PIN1 (8 μg), the psAX2 (6 μg) packaging plasmid, and the pMD2G (2 μg) envelope plasmid (4:3:1) were transfected into HEK293 T cells with 10 cm culture dish using Lipofectamine 3000 (Invitrogen, Carlsbad, CA, USA) to obtain lentiviral supernatant. After 48 h incubation, 2 μg/mL puromycin (Sangon Biotech, Shanghai, China) was added for two weeks to select for stable knockdown cells. To generate PIN1 stably overexpressing cells, HA-tagged PIN1 was cloned into a pCDH-CMV-MCS-EF1 vector (System Biosciences, Palo Alto, CA, USA). All cell transfections were performed using Lipofectamine 3000 (Invitrogen, Carlsbad, CA, USA) according to the manufacturer’s instructions.

### 4.6. Total RNA Extraction and Quantitative Real-Time PCR (qRT-PCR)

Whole tumor cell RNA was extracted with TRIzol reagent (Sigma, St. Louis, MO, USA, T9424)/chloroform, purified using the PureLink RNA Minikit (Life Technologies, Carlsbad, CA, USA), assessed for quality and quantity using absorption measurements, and then a total of 500 ng RNA was used for a double round of cDNA synthesis. The expression status of the candidate genes and GAPDH was determined by quantitative real-time PCR using an ABI 7900HT Real-Time PCR system (Applied Biosystems, Bedford, MA, USA). Comparative data analysis was performed via the ΔΔCt method using the PCR Array Data analysis web portal to determine relative expression differences between the comparison groups. All reactions were run in triplicate, and primer sequences are listed in Supplementary Table S1.

### 4.7. Protein Extraction and Western Blot Analysis

Cells were washed twice with ice-cold PBS and lysed in RIPA buffer for 20 min. Cell debris was removed by centrifugation at 12,000 rpm for 15 min at 4 °C. Total protein lysate (20–40 μg) was subjected to electrophoresis in a denaturing 10% SDS-polyacrylamide gel and then transferred to a membrane for subsequent blotting with specific antibodies against GAPDH, PIN1, and NRF2. PIN1 antibodies (diluted 1:1000) were purchased from Abcam (ab38933) and NRF2 (diluted 1:1000) were obtained from Abcam (ab62352). Western blotting was carried out as previously described in details [34].

### 4.8. Statistical Analysis

The data are presented as the mean ± standard deviation (SD). Experiments were repeated at least three times. Two-tailed unpaired Student’s *t*-tests and one-way analysis of variance were used to evaluate the differences. Spearman correlation analysis was used to determine the correlation between stroma proportion and PIN1 expression level. SPSS version 16.0 software (Chicago, IL, USA).) was used for data analyses. Differences were considered significant at * *p* < 0.05; ** *p* < 0.01; and *** *p* < 0.001.

## Data Availability

Not applicable.

## Supplementary Materials

The supporting information can be downloaded at: https://www.mdpi.com/article/10.3390/ijms24043476/s1.
