# Supplementary material for: Mechanical Regulation of Redox Balance via the Induction of the PIN1/NRF2/ARE Axis in Pancreatic Cancer
Source: Int J Mol Sci. 2023 Feb 9;24(4):3476. doi: 10.3390/ijms24043476 (PMC9961122; doi:10.3390/ijms24043476)
Supplement: Supplementary file 1 [file ijms-24-03476-s001.zip › ijms-2206449-supplementary.pdf]

Supplementary Table S1：The sequences of primers for qRT-PCR

	Sequence (5’-3’)
PIN1 Forward	GAGAAGATCACCCGGACCAAGGAG
PIN1 Reverse	GAGAAGATCACCCGGACCAAGGAG
HMOX forward	AAGACTGCGTTCCTGCTCAAC
HMOX reverse	AAAGCCCTACAGCAACTGTCG
GCLC Forward	GTGGTACTGCTCACCAGAGTG
GCLC Reverse	AGCTCCGTGCTGTTCTGGGCCTT
GCLM Forward	ATCTTGCCTCCTGCTGTGTGATGC
GCLM Reverse	CAATGACCGAATACCGCAGTAGCC
ME1 Forward	CCTCACTACTGCTGAGGTTATAGC
ME1 Reverse	CGGTTCAGGATAAACTGTGGCTG
NQO1 Forward	CGGAGTAAGAAGGCAGTGCTTTC
NQO1 Reverse	TCTGCTGGAGTGTGCCCAATGCT
GAPDH forward	GGAGCGAGATCCCTCCAAAAT
GAPDH reverse	GGCTGTTGTCATACTTCTCATGG
